# Impact of an obesogenic diet program on bone densitometry, micro architecture and metabolism in male rat

**DOI:** 10.1186/1476-511X-11-91

**Published:** 2012-07-10

**Authors:** Maude Gerbaix, Lore Metz, Fabrice Mac-Way, Cédric Lavet, Christelle Guillet, Stéphane Walrand, Aurélie Masgrau, Marie-Thérèse Linossier, Laurence Vico, Courteix Daniel

**Affiliations:** 1 Laboratoire des Adaptations Métaboliques à l’Exercice en conditions Physiologiques et Pathologiques (AME2P), Clermont Université, Université Blaise Pascal, EA 3533, BP 80026, F-63171, Aubière Cedex, France; 2 Laboratoire de Biologie Intégrative du Tissu Osseux, Inserm U1059, Université de Lyon, 42023 cedex, Saint-Etienne, France; 3 Centre de recherche du CHUQ, L’Hôtel-Dieu de Québec, Université Laval, Québec, Canada; 4 INRA, UMR 1019, CRNH Auvergne, F-63000, Clermont-Ferrand, France

## Abstract

**Background:**

The relationships between fat mass and bone tissue are complex and not fully elucidated. A high-fat/high-sucrose diet has been shown to induce harmful effects on bone micro architecture and bone biomechanics of rat. When such diet leads to obesity, it may induce an improvement of biomechanical bone parameters in rodent.

Here, we examined the impact of a high-fat/high-sucrose diet on the body composition and its resulting effects on bone density and structure in male rats. Forty three Wistar rats aged 7 months were split into 3 groups: 1 sacrificed before diet (BD, n = 14); 1 subjected to 16 weeks of high-fat/high-sucrose diet (HF/HS, n = 14); 1 subjected to standard diet (Control, n = 15). Abdominal circumference and insulin sensitivity were measured and visceral fat mass was weighed. The bone mineral density (BMD) was analyzed at the whole body and tibia by densitometry. Microcomputed tomography and histomorphometric analysis were performed at L2 vertebrae and tibia to study the trabecular and cortical bone structures and the bone cell activities. Osteocalcin and CTX levels were performed to assess the relative balance of the bone formation and resorption. Differences between groups have been tested with an ANOVA with subsequent Scheffe post-hoc test. An ANCOVA with global mass and global fat as covariates was used to determine the potential implication of the resulting mechanical loading on bone.

**Results:**

The HF/HS group had higher body mass, fat masses and abdominal circumference and developed an impaired glucose tolerance (p < 0.001). Whole body bone mass (p < 0.001) and BMD (p < 0.05) were higher in HF/HS group vs. Control group. The trabecular thickness at vertebrae and the cortical porosity of tibia were improved (p < 0.05) in HF/HS group. Bone formation was predominant in HF/HS group while an unbalance bone favoring bone resorption was observed in the controls. The HF/HS and Control groups had higher total and abdominal fat masses and altered bone parameters vs. BD group.

**Conclusions:**

The HF/HS diet had induced obesity and impaired glucose tolerance. These changes resulted in an improvement of quantitative, qualitative and metabolic bone parameters. The fat mass increase partly explained these observations.

## Background

A sedentary life-style as well as high energy food consumption contribute to the burden of obesity. Fat mass accumulation induces changes in body composition that affect the bone health. The effect of body mass on the skeleton remains controversial although numerous studies in the literature exist concerning obese subjects [1-3]. Some studies have shown that obese adolescents have higher bone mineral density (BMD) than their normal-weight controls [4] and that obesity offers some protection against osteoporosis [5]. Alternatively, other studies have shown an inverse relationship between bone mass and fat mass after adjustment for body weight [6]. In animals, the effects of high fat/high sucrose diet (HF/HS) on bone health are also controversial [7-9]. Since the experimental methods are quite disparate between studies, it is not surprising that findings are discordant as well. These discrepancies could be explained by the different animal characteristics (gender, age and race), the duration and the quality of the diet used. But the most important factor that could also influence the results is the ability of the diet to induce or not obesity. By inducing mechanical load on the skeleton, the excess of fat mass is strongly linked to bone tissue. Some studies reported improvements of biomechanics and microarchitecture of femur in rodents previously fed with diets inducing obesity [9,10]. Bramhabhatt et al. [9] showed that the same HF/HS diet could cause a divergent body weight response, some being obese and others not. They suggested that obese rats had favorably adapted their bone tissues and improved their biomechanical properties compared to rats “resistant” to the diet. Such results show that obesity must be taken into account when performing relevant comparisons between studies. Indeed, when HF or HF/HS diet does not induce obesity, the rats develop lower bone mineral density or worst bone mechanical properties [7,8,11]. There is a consistent positive association between body mass and bone mass. Conversely, weight loss is linked to a concomitant accelerated loss of bone mass [12-14]. Dietary factors are known to influence BMD. Several studies have examined the association between the type of dietary fat and BMD in humans but again the results are conflicting. Thus, saturated fatty acid intake was found to correlate inversely to the BMD in men and women in NHANES cohort study [15] yet Brownbill and Ilich [16] did not find any association in a study concerning white post-menopausal women.

All these divergences probably derive from the complex relationship existing between adipose and bone tissues. Until now, only few studies have analyzed the effect of an obesogenic diet on bone characteristics in male rats and none of them have assessed the cellular activity of bone remodeling.

Therefore, the purpose of the present study was to investigate the bone cellular activity, density and micro architecture response to body composition changes induced by an HF/HS diet in male rat.

## Methods

### Animal care and experimental diet

All experimental designs and procedures were made in accordance to the current legislation on animal experience in France and were approved by the ethical committee for animal experimentation (CREEA Auvergne, CE1-09). Forty-three 6-month old Wistar male rats (CERJ Janvier®, Le Genest Saint-Isle, France) were individually housed in a temperature-controlled room (20-22°C) and a reversed light–dark cycle (light on 20 h00-08 h00) was maintained. After one month of acclimatization with a standard rodent diet, animals (aged of 7 months) were randomized into three groups: 16 weeks of high-fat/high-sucrose diet group (HF/HS, n = 14); 16 weeks of standard diet group (Control, n = 15) and 1 group sacrificed before the diet (BD, n = 14). The rats had free access to water. The composition of the diets and repartition of types of fatty acids are given Table 1. HF/HS diet was mainly enriched in sucrose, saturated fatty acids and cholesterol (provided by lard) resulting in omega6/omega3 ratio higher than in standard diet (7.16 vs. 5.63). Although the diet differed in dietary composition, each provided similar daily amounts of protein, cellulose, vitamins and minerals to the animals based on the two groups being fed the same daily caloric intake. In order to control that all groups consumed equal amounts of calories each day, the diets were prepared in individual ramekins and removed daily. Finally, ninety five kcal of food per day were given to all rats.

**Table 1:** Composition of the experimental diets and type of fatty acid

	**Standard Diet**					**High Fat/High Sucrose Diet**				
**Ingredients**	**(g/kg)**	**Carbohydrates**	**Protein**	**Lipid from animal**	**Lipid from plant**	**(g/kg)**	**Carbohydrates**	**Protein**	**Lipid from animal**	**Lipid from plant**
Casein	170	-	170	-	-	204	-	204	-	-
Mineral mix	45	-	-	-	-	54	-	-	-	-
Vitamin mix	10	-	-	-	-	12	-	-	-	-
Cornstrach	670	670	-	-	-	222	222	-	-	-
Sucrose	0	-	-	-	-	222	222	-	-	-
peanut oil	30	-	-	-	30	35	-	-	-	35
rapeseed oil	30	-	-	-	30	35	-	-	-	35
Lard	0	-	-	-	-	164	-	-	164	-
Cellulose	45	-	-	-	-	52	-	-	-	-
Total	1000	670	170	-	60	1000	444	204	164	70
Energy (kcal/kg)	3900	2680	680	-	540	4698	1776	816	1476	630
% kcal		69	17	-	14		38	17	31	13
Saturated fatty acid (% of total lipids)				12.6		32.9				
Monounsaturated fatty acid (% of total lipids)				55.1		49.3				
Polyunsaturated fatty acid (% of total lipids)				32.3		17.8				

### Diet follow-up

Rats were weighed every week to record their body mass. Total body composition was assessed every three weeks by DXA on a sample of 20 rats (HF/HS, n = 10; Control, n = 10).

### Total body composition, central Fat mass and bone mineral density by DXA

A Hologic QDR 4500 device was used with an internal adapted collimator for small animal measurements (Hologic QDR Software for Windows XP version, Copyright© 1986–2002 Hologic Inc.). The rats were anaesthetized before measurements. Anesthesia consisted on an intra-peritoneal injection of a solution of Acepromazine Vetranquil® (0.5 ml/kg of body weight) and Ketamine Imalgène® (0.75 ml/kg of body weight). After anesthesia, rats were positioned ventrally on a reference film to reproduce the position. One week before sacrifice, the total body composition and bone mineral density of all animals were assessed by DXA using specific small animal body composition software. DXA-derived lean tissue mass was used as a surrogate of muscle mass. The coefficients of variation (CV) were determined for these parameters from six repeated measurements with repositioning on eight animals. CVs were 1.20%, 4.19% and 0.81% for global mass, global fat and bone mineral density respectively. Central Fat Mass (CFM) which method and validation has been published recently [17] was assessed . Briefly, CFM can be distinguished using DXA by identifying it as a specific region of interest within the analysis program. Fat mass from this region was strongly correlated with weighted visceral fat mass (r = 0.094; p < 0.001) and had been validated to be a useful predictor of visceral fat mass.

### Dissection of rats

Rats were fasted for 12 hours before sacrifice. They were euthanized by decapitation under isoflurane anesthesia. Visceral fat mass was assessed by weighing the total perirenal and peri-epididymal adipose tissues. The weights of these two tissues were combined to form the *ex-vivo Fat Mass*. Right tibia and L2 vertebrae were removed for bone microarchitecture and histomophometric analyses. Left tibia was removed for densitometric analyses.

### Abdominal circumference measurement (AC)

Abdominal circumference (AC) was assessed on all rats on the largest circumference of the rat abdomen using a plastic non extensible measuring tape (Rollfix, Hoechstmass®, Germany) with an accuracy of 0.1 cm. Rats were placed in ventral position. It was shown that the AC measure could be a useful biometric technique for assessing in-vivo abdominal fat mass storage in fat rats [17]. The CV for AC measures (2.69%) was determined following three analyses on 13 rats. The same operator repositioned the measuring tape three times.

### Oral glucose tolerance tests (OGTT)

All rats were subjected to an OGTT one week before sacrifice. After 13 hours fasting, blood samples were collected from the tail vein using heparinized capillary tubes. The rats were then given a glucose load solution by gavage (1 g/kg of body weight) and vein tail blood was collected 15, 30, 60, 90 and 120 minutes later. The blood samples were centrifuged at 13 000 g for three minutes to obtain the plasma which was stored at −80°C and subsequently assayed for glucose (bioMérieux® SA, Marcy-l’Etoile, France) and insulin (Millipore Corporation, Billerica, MA, U.S.A.). The glucose and insulin responses during the OGTT were computed from the area under the curve (AUC) using the trapezoidal method [18].

### Biochemical assays

Blood samples were collected right after decapitation. The blood samples were immediately centrifuged and plasma was stored at −80°C until measurements. All biochemical measures were assessed in duplicate. Lipids profile of all rats (total cholesterol, HDL cholesterol, triglycerides, non-esterified fatty acids (NEFA),) was measured in plasma samples by using an automated analyser (Konelab 20, Thermo Electron Corporation). Chemicals were obtained from Thermo Fischer (Thermo Fisher Scientific, Vantaa, Finland). The intra assay coefficients of variations were 2.89%, 2.3%, 2.07% and 3.01% for total cholesterol, HDL cholesterol, triglycerides and NEFA respectively. The inter assay coefficients of variation were 4.04%, 4%, 4.5% and 4.2% for total cholesterol, HDL cholesterol, triglyceride and NEFA respectively. LDL cholesterol level had been determined using Freidwald formula (LDL cholesterol = Total Cholesterol - HDL cholestérol – (triglycerides/5)).

Hormonal levels of insulin, leptin and adiponectin were measured on the plasma of each rat by ELISA kit (Millipore Corporation Headquarters, Billerica, MA, U.S.A.).

The intra-assay coefficients of variation were 3.22%, 2.49% and 1.96% for insulin, leptin and adiponectine respectively. The inter assay coefficient of variations were 6.95%, 3.93% and 8.44% for insulin, leptin and adiponectine respectively.

The bone turnover markers have been measured on plasma of eight rats per group. Bone formation was measured by serum level of osteocalcin using Elisa essays (Rat-MID™ Osteocalcin EIA IDS, UK) and bone resorption by serum level C-terminal telopeptide of type I collagen (CTX) using an Elisa essays (Kit Rat-Laps™ EIA IDS, UK). The intra-assay coefficients of variation were 9.2% and 3.6% for CTX and osteocalcin respectively. The inter assay coefficient of variations were 14.8% and 6.6% for CTX and osteocalcin respectively. In order to assess the relative balance of the formation and resorption, we calculated the bone uncoupling index (UI) [19]. Using the BD group values as reference data, z scores of formation and resorption markers were calculated for each rats. Then, the UI was calculated as the average of the z score for the bone formation marker minus the bone resorption marker. A positive UI indicates that bone formation was predominant while and a negative UI indicates an imbalance favouring resorption [20].

### Bone histomorphometry

Bone labeling of 7 rats per groups was performed by intra-peritoneal injection of tetracycline (30 mg/kg of body weight) 7 days and 1 day before death. After 48 hours of fixation with formol (10%) and dehydration in acetone, the right tibia and the L2 vertebrae of seven rats per groups were embedded in methylmethacrylate at a low temperature with known techniques (27). The central plane of metaphyses of the tibia was sliced frontally with a microtome (Reichert-Jung Polycut, Heidelberg, Germany). Nine micrometers thick slices were retrieved for Goldern’s trichrome and Tartrate-resistant Acid Phosphatase (TRAcP) (5 slices each) and Toludin blue (2 slices) colorations. Dynamic parameters were evaluated from five unstanded slices of 12 μm. The following parameters were measured in the secondary spongiosa according to the ASBMR histomorphometry nomenclature (Parfitt et al., 1987) using an automatic image analyzer (BIOCOM, Lyon, France): BV/TV, Tb.Th, and osteoid surface (OS/BS, %). TRAcP staining permitted the measurements of osteoclastic surfaces activity (Oc.S/BS) and osteoclast number (N.Oc). Histodynamic parameters were determined under UV light: mineral apposition rate (MAR, μm/day), single labeled surface (sLS/BS, %), and double-labeled surface (dLS/BS, %). Mineralizing surface per bone surface (MS/BS, %) was calculated by adding dLS/BS and one-half sLS/BS. Bone formation rate (BFR/BS, µm3/µm2/day) was calculated as the product of MS/BS and MAR. The aforementioned parameters of bone resorption and formation were measured with a semiautomatic system consisting of a digitizing table (Summasketch-Summagraphics, Paris, France) connected to a personal computer and a Reichert Polyvar microscope equipped with a drawing system (Camera Lucida; Reichert-Jung Polyvar).

### High resolution micro tomography (μCT)

Right tibia and L2 of all rats were scanned ex vivo with high-resolution μCT (VivaCT40, Scanco Medical,Bassersdorf, Switzerland). Trabecular network is qualified by the plate-rod characteristic of the structure (Structure Model Index), the geometric degree of anisotropy and connectivity density. The tibia secondary spongiosa was scanned within the metaphysis below the growth plate and the cortical bone of tibia was scanned in the diaphysis. The L2 secondary spongiosa was scanned between the two growth plates. Exactly, 645 slices were set for total acquisition in both tibia and vertebrae. The cortical ROI of tibia identified 100 slices under the end of trabecular area in order to avoid secondary spongiosa. Data were acquired at 55 keV, with a 10 μm cubic resolution. Three-dimensional reconstructions were generated using the following parameters: Sigma: 1.2, Support: 2, Threshold: 250 for trabecular bone, and 280 for cortical bone. The structural parameters of trabecular bone: bone volume fraction (BV/TV), trabecular thickness (Tb.Th), trabecular number (Tb.N), trabecular separation (Tb.Sp), structure model index (SMI), connection density (Conn.D.) and degree of anisotropy (DA) were generated from a set of 250 slides [21], cortical thickness and cortical bone volume fraction (BV/TV) were calculated by integrating the value on each transverse section of a set of 100 slices chosen in the midshaft area. Cortical porosity was calculated as follows: Cortical porosity = (1 – (BV/TV) *100).

### Ex-vivo densitometry

Densitometric parameters of the left tibia were assessed by a densitometer designed for small animal body composition (PIXImus, Lunar® corporation) with a spacial resolution of 0.18 mm. Total bone mineral density, bone mineral content and bone area have been assessed.

### Statistical method

The Gaussian distribution for each parameter was assessed by a Shapiro-Wilk test. In case of non-normal distribution, the data were log-transformed for analyses. In order to assess time evolution, a repeated measures ANOVA was performed. Differences between groups have been tested with an ANOVA with subsequent Scheffe post-hoc test. An ANCOVA with global mass and global fat as covariates was used to determine the potential implication of the resulting mechanical loading on bone. P values of less than 0.05 were considered to be significant. * p < 0.05 ** p < 0.01 *** p < 0.001. Data are presented as mean ± SD excepted biochemical data (presented as mean ± SE). Analysis was carried out using SPSS Advanced Statistics software (version 17).

## Results

### Body weight and body composition during diet period

**Figure 1:**
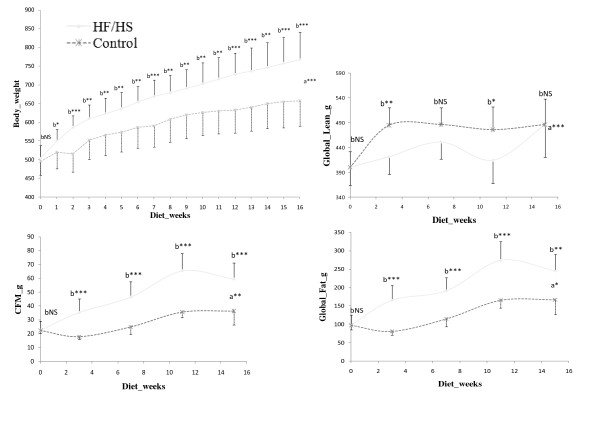
**Weight, Central Fat Mass, Global lean and Global lean and Global Fat progression during diet.** a = interactions between groups. b = differences between groups at each measure. CFM = Central Fat Mass. Weight follow has been assessed on each rat. Central Fat Mass and global fat have been assessed on 10 rats per group.

Figure 1 shows body weight, lean mass and fat masses follow-up by DXA during the diet period. From the first week of diet, rats fed with HF/HS showed significantly higher weight gain than the Control group. HF/HS group had significantly higher global fat, central fat mass and global fat percentage from the first measure (3 weeks of diet) (p < 0.01). It is also noticeable that Control group had significant higher lean mass at 3 weeks and 11 weeks of the diet period but these differences disappeared at the end of the diet (week 15).

### Final body composition, visceral fat, central fat mass and abdominal circumference

Final body composition, visceral fat, central fat mass and abdominal circumference values are given Table 2. HF/HS group displayed significantly higher global mass, global fat and bone mass (p < 0.001) than Control group. HF/HS group had also higher global fat percentage, abdominal fat mass, central fat mass and ex vivo fat mass than Control group (p < 0.001). Lean mass was not different between HF/HS group and Control group. Furthermore, comparisons to BD group showed that HF/HS and Control groups had higher total mass (p < 0.001) with higher global fat (p < 0.001), higher lean mass (p < 0.001) and a higher bone mass (p < 0.001) than BD group. The HF/HS and Control groups had also higher values of global fat percentage, ex vivo fat mass, abdominal fat mass and central fat mass (p < 0.001) compared with BD group.

**Table 2:** Body Composition. Obesity Parameters. Lipid and hormonal profiles

		**BD**^**a**^		**HF/HS**^**b**^		**Control**^**c**^		**ANOVA**	**Post-hoc**
		**Mean**	**SD_SE**	**Mean**	**SD_SE**	**Mean**	**SD_SE**	**p**	
Body Composition	GLOBAL MASS (g)	495.5	33.3	729.7	67.0	627.1	62.8	0.000	a < c < b
	GLOBAL FAT (g)	93.9	25.4	248.1	59.5	159.0	39.2	0.000	a < c < b
	GLOBAL LEAN (g)	386.8	31.3	461.9	31.8	450.0	56.16	0.000	a < c.b
	GLOBAL BMC (g)	14.75	0.99	19.71	1.61	17.96	1.90	0.000	a < c < b
Obesity Parameters	GLOBAL PFAT (%)	18.9	4.5	33.7	5.4	25.3	5.5	0.000	a < c < b
	AC (cm)	21.2	0.7	26.5	1.4	23.4	1.3	0.000	a < c < b
	CFM (g)	22.0	7.6	64.1	15.3	34.6	9.3	0.000	a < c < b
	Perirenal AT (g)	10.6	3.4	34.5	7.5	12.6	3.4	0.000	a.c < b
	Peri-epididymal AT (g)	9.2	2.7	25.5	5.6	16.3	3.5	0.000	a < c < b
	*ex vivo* Fat Mass (g)	19.8	5.8	60.0	12.1	28.9	6.4	0.000	a < c < b
Lipid Profile	TRIGLY (g/l)	0.97	0.09	0.64	0.03	1.12	0.09	0.000	b < a.c
	Total CHOL (g/l)	0.94	0.09	0.64	0.03	1.10	0.05	0.075	
	HDL CHOL (g/l)	0.48	0.04	0.49	0.04	0.62	0.03	0.013	a.b < c
	LDL CHOL (g/l)	0.27	0.07	0.28	0.03	0.26	0.03	0.258	
	NEFA (mmol/l)	0.49	0.03	0.28	0.01	0.75	0.08	0.000	b < a < c
Hormonal Profile	INSULIN (ng/ml)	0.475	0.039	0.633	0.080	0.765	0.052	0.001	a < c
	LEPTIN (ng/ml)	4.300	0.448	19.144	0.915	10.692	1.246	0.000	a < c < b
	ADIPONECTIN (μgm/l)	11.292	0.590	12.563	0.762	12.240	0.689	0.443	

Body composition and obesity parameters data are presented as mean ± SD. Lipid and hormonal profiles are presented as mean ± SE. AC = Abdominal Circumference. CFM = Central Fat Mass. AT = Adipose Tissues. CHOL = Cholesterol. NEFA = Non-Esterified Fatty Acids. BD n = 14. HF/HS n = 14. Control n = 15.

### Glucose tolerance tests (OGTT)

**Figure 2:**
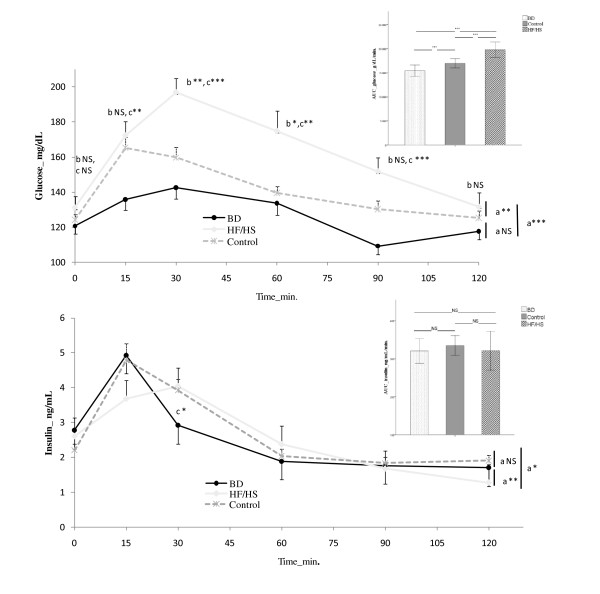
**Glycemia and Insulinemia during oral glucose tolerance test and areas under the curve (AUC) for glucose and insulin.** a = interactions between groups. b = difference over times between HF/HS group and Control group. c = difference over time between HF/HS group and BD group. BD n = 14, HF/HS n = 14, Control n = 15.

Glucose and insulin evolution during OGTT and their AUC are displayed on Figure 2. There was no difference on fasting plasma glucose and insulin levels between groups. AUC for glucose was significantly higher in HF/HS group compared with both Control and BD groups (p < 0.001). AUC for insulin was not different between groups. Regarding time effect, the glucose levels were significantly higher in HF/HS group at 30, 60 and 90 min after glucose administration versus Control group. No difference was found between Control and BD group.

### Serum analysis

Lipid and hormonal profiles are given in Table 2. Results from lipid profile showed that HF/HS group had significantly lower triglycerides (p < 0.001), HDL cholesterol (p < 0.05) and NEFA (p < 0.001) levels compared with Control group. The HF/HS group had also significantly lower triglycerides (p < 0.001) and NEFA (p < 0.001) levels than BD group. The control group had higher HDL (p < 0.05) and NEFA (p < 0.001) levels than BD group. Results from hormonal profile showed that the HF/HS group had higher leptin level than the Control group (p < 0.001). The insulin and leptin levels were higher in Control group compared to BD group (p < 0.001). The adiponectin level was not different between groups.

### Bone investigation

**Figure 3:**
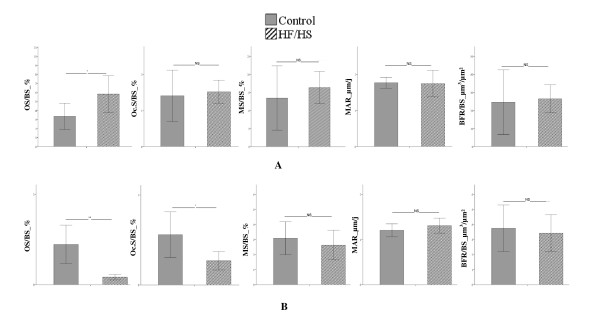
**Histomorphometric parameters of tibia (A) and L2 vertebrae (B).** OS = osteoid surface, BS = bone surface, BFR = bone formation rate, Oc.S = osteoclastic surface, MAR = mineral apposition rate, MS = mineralizing surface. Values are means ± SD. BD n = 7, HF/HS n = 7, Control n = 7.

**Figure 4:**
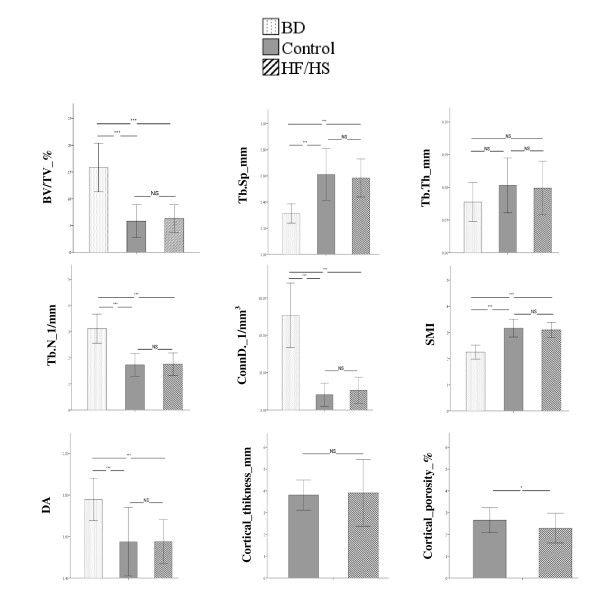
**Tibia trabecular and cortical micro architecture, BV/TV = relative bone volume, Tb.Th = trabecular thickness, Tb.Sp = trabecular separation Tb.**Th = trabecular thickness, Tb.N = trabecular number, ConnD. = connectivity density, SMI = structure model index. DA = degree of anisotropy. Values are means ± SD. BD n = 14, HF/HS n = 14, Control n = 15.

The whole body BMD was significantly greater in HF/HS group compared to Control group (0.186 ± 0.008 g/cm² vs. 0.178 ± 0.009 g/cm²; p < 0.05) and BMD of tibia tended to be higher in HF/HS group versus Control group although no statistically significant (0.214 ± 0.010 g/cm² vs. 0.205 ± 0.017 g/cm²; p = 0.065). Figure 3 displays the trabecular histomorphometric parameters of tibia (A) and vertebrae (B). The bone histodynamic and resorption parameters were not different in both tibia and vertebrae between HF/HS group and Control group. The osteoid surface was significantly higher in tibia (p < 0.05) and significantly lower in vertebrae (p < 0.01) in HF/HS group versus Control group. The bone turnover markers analysis revealed that the osteocalcin level was significantly reduced in Control group versus BD group (106.2 ± 8.4 ng/mL vs. 161.7 ± 16.8 ng/mL, p < 0.05) while no differences were observed between HF/HS group and both BD and Control groups (HF/HS = 119.7 ± 11.9 ng/mL; BD = 161.7 ± 16.8 ng/mL; Control =106.2 ± 8.4 ng/mL, NS). CTX level were not significantly different between the three groups (BD = 14.6 ±0.7; HF/HS = 11.9 ± 0.6; Control = 13.2 ± 1.8; NS). The bone uncoupling index in HF/HS group was positive (0.37 ± 0.35) indicating that there was an unbalanced remodeling favouring bone formation whereas the bone uncoupling index was negative in Control group (−0.70 ± 0.69). Figure 4 displays the trabecular and cortical micro-architecture parameters of tibia. Trabecular parameters were not different between HF/HS and Control groups. Results from cortical parameters showed that the HF/HS group had significantly lower cortical porosity than Control group (p < 0.05). When compared to the BD group, HF/HS and Control groups had significantly lower BV/TV, Tb.N, ConnD. and degree of anisotropy (p < 0.001) and displayed significantly higher Tb.Sp and SMI (p < 0.001). The results of micro-architecture of vertebrae revealed that the HF/HS group had larger Tb.Th than Control group (0.099 ± 0.008 mm vs. 0.090 ± 0.007 mm; p < 0.01). When compared to the BD group, the HF/HS group had a higher SMI (1.18 ± 0.28 vs. 0.77 ± 0.33; p < 0.05) and a lower degree of anisotropy (1.69 ± 0.11 vs. 1.9 ± 0.08; p < 0.001). Results from comparisons between the Control group and the BD group showed that the Control group had a significant lower BV/TV (0.22 ± 0.07 vs. 0.29 ± 0.04; p < 0.05), Tb.N (2.82 ± 0.61 1/mm vs. 3.37 ± 0.33 1/mm; p < 0.05), ConnD. (32.26 ± 13.59 1/mm^3^ vs. 49.5 ± 10.20 1/mm^3^; p < 0.01) and degree of anisotropy (1.75 ± 0.16 vs. 1.90 ± 0.08; p < 0.001). The Control group also displayed a higher Tb.Sp (0.36 ± 0.16 mm vs. 0.26 ± 0.03 mm; p < 0.05) and SMI (1.19 ± 0.43 vs. 0.77 ± 0.33; p < 0.05) than the BD group.

After adjusting for total mass and fat mass, the significant differences in bone parameters between HF/HS group and Control group remained unchanged in vertebrae but disappeared in the tibia.

## Discussion

The main findings of the present study show an improvement of bone tissue in obese rats. The obesity induced by the HF/HS diet was characterized by an increase in total, visceral and central fat masses without change of lean mass. As expected, the ageing process occurred during the period of investigation has produced deleterious effects on bone tissue in both Control and HF/HS diet groups. However, compared to the Control diet, the HF/HS diet has induced a higher total bone mineral density, a lower cortical porosity and a bone remodelling favouring bone formation as shown by the positive uncoupling index and the increase in bone osteoid surface on tibia. The vertebrae bone micro architecture improved as well (increase in trabecular thickness). Furthermore, it is relevant to note that bone tissue response to obesity was different according to the bone site analyzed.

We chose to control the food intake so that all groups consumed equal amounts of calories each day, which ensured that any diet effect was due to the macronutrient composition and not due to differences of energy consumed by each group. This method guaranteed that accumulation of fat was mainly due to supplementation of saturated fatty acid and/or sucrose. In a previous study, Dourmashkin et al. have concluded that both HF and HS diets were able to increase body weight and fat mass [22]. In the present study, we could state that increase in body weight and fat accumulation were due to both sucrose and saturated fatty acids.

The lipid profile analysis showed that HF/HS diet had induced significantly lower triglycerides, HDL cholesterol and free fatty acid levels compared to the standard diet. These results could point out a lesser lipid mobilization in obese rats. Whereas most studies have shown that high fat diet may induce an increase of these lipids levels [23,24], other studies reported that lipids levels were unchanged or even decreased [25,26].

Result from oral glucose tolerance test showed that HF/HS group had a higher area under the curve for glucose but not for insulin. For the same insulin secretion, the glucose “peak” response was significantly higher and the return time to the basal level was longer in HF/HS group compared to control. These results suggest that the HF/HS diet group was not able to adequately respond to the elevated glucose levels as their insulin concentrations were similar to those of the control group that had significantly lower glucose levels. Although the HF/HS diet rats may have developed an impaired glucose tolerance, there was no evidence that HF/HS rats had developed an insulin resistance. Unfortunately, the present results are not in accordance with those observed currently in human. Contrary to the human response, the animal response seems to be more complex to analyze and interpret.

Insulin resistance and type2 diabetes without obesity are associated with low bone mineral density and increased risk of fracture in both humans and animals [27,28]. In the present study, the improvement of bone quality could be also explained by the absence of insulin resistance in obese rats.

In previous studies using female rats, the HF/HS diet has been shown to induce adverse effects on bone health especially on cortical bone morphology and bone mineral content of vertebrae [7,8]. A recent study [29] showed that female and male rats responded differently to a diet-induced obesity. A possible explanation for this difference could be the gender-specific changes in leptin or ghrelin under the HF feeding [30]. Indeed, Harris et al. demonstrated that male mice fed with high fat diet had developed a leptin resistance in response to an injection of exogenous leptin whereas female mice remained leptin sensitive. They concluded that the development of leptin resistance in mice fed with HF diet is dependent upon the gender. Furthermore, one must consider that using female animals implicate additional hormonal effects of estrogen on bone metabolism, morphology and biomechanics [31].

Bone tissue is a modeling structure subjected to many intrinsic and extrinsic factors. On one hand, the benefit of a diet-induced obesity on bone could be explained by the mechanical loading caused by the excess of body mass. On the other hand, adipose tissue is known to be an active endocrine organ secreting many biological active molecules such as leptin and adiponectin. In our study the adiponectin level was not affected by the obesogenic diet. However, the leptin level was almost twice in obese group compared to the control group. Leptin effects on bone are complex depending of its central or peripherial pathways [32] and on its serum concentration [33]. The central effect of leptin favors resorption through the sympathetic nervous system [34]. A high bone mass phenotype has been observed in leptin-deficient ob/ob mice which could not be explained by their adiposity since mice lacking adipocytes displays the same phenotype [35]. In our study, it is possible that obese rats would have developed a leptin endogenous resistance which has been shown in obese mice fed with high fat diet [36]. Leptin resistance increases with fat mass storage and age [37,38]. This phenomenon could then explain the difference of results observed between our study and those who worked on leaner and younger rats [7,8,11]. Leptin has also a direct anabolic effect within the bone micro-environment by stimulating the differentiation of bone marrow mesenchymal stem cells into osteoblastic cell lineage [39]. These elements could explain a possible implication of leptin in the higher bone mass observed in obese rats in our study.

After adjustments for total and fat mass, the differences in the bone parameters observed between obese and control rats remained unchanged in vertebrae but became not significant in the tibia. This suggests a site-dependent response possibly due to the increase of mechanical loading provided by the excess of fat mass. While fat mass could partly explain the improvement of bone quality of the tibia (a loaded bone site), vertebrae (a non loaded bone site) was not affected by this factor. These results are in concordance with previous studies, which found that obesity and functional load, affected regional bone mineral density in a different manner [40,41]. These results also suggest that mechanical loading is not the only parameter contributing to the improvement of bone health in obese rats. Other parameters are also implicated among which, leptin seems to play an important role.

## Conclusions

While obesity is most commonly associated with metabolic complications, the present study showed that an obesogenic diet has favorable effects on bone tissue. The specific isoenergetic HF/HS diet used within the current study had induced obesity but no insulin resistance. This form of obesity improved cortical and trabecular bone parameters in adult male rats. These adaptations were partly due to the increase in body mass-induced mechanical load, which affected bone tissue differently according to the analyzed site.

## Competing interests

The authors have no conflict of interest.

## Authors’ contributions

MG has participated in the investigation as PhD student responsible of the protocol; intervention on animals, analysis and interpretation of data, drafting the manuscript. LM, FMW, LMT,CG, SW, AM, LV, DC have contributed to conception and design, intervention on animals, acquisition of data. LM has taken responsibilities in the housing protocol and measurements. DC had responsibility of the design, analysis and interpretation of data and participated in drafting the manuscript, revising it critically for intellectual content and has given final approval of the version to be published. All authors read and have approved the final manuscript.
